# Dimensional Stability and Compressive Resistance of Three Interocclusal Record Materials: A Comparative In Vitro Study

**DOI:** 10.7759/cureus.71718

**Published:** 2024-10-17

**Authors:** Arjun SL, Manoharan PS, Rajkumar Eugene, Ponsekar Abraham Anandapandian

**Affiliations:** 1 Prosthodontics, Thai Moogambigai Dental College and Hospital, Chennai, IND; 2 Prosthodontics, Indira Gandhi Institute of Dental Sciences, Pondicherry, IND

**Keywords:** bonabite, bona-bite, compressive resistance, dimensional stability, interocclusal record materials, o-bite, polyvinyl siloxane, prosthetic accuracy, ramitec

## Abstract

Background: Accurate interocclusal records are crucial for a successful prosthetic treatment as they ensure the correct replication of a patient’s jaw movements and relationships. The dimensional stability and compressive resistance of interocclusal record materials are essential factors influencing the quality of the final prosthesis. This study aims to evaluate and compare the dimensional stability and compressive resistance of three commonly used interocclusal recording materials: Ramitec (polyether; 3M ESPE, St. Paul, MN), Bona-Bite (polyvinyl siloxane; DMP Dental, Markopoulo, Greece), and O-Bite (polyvinyl siloxane; DMG Dental, Hamburg, Germany).

Methodology: An in vitro study was conducted using 108 samples prepared from three interocclusal record materials: Ramitec, Bona-Bite, and O-Bite, with each material tested at three different thicknesses (2, 4, and 6mm). A master stainless steel die was used to create the samples. The compressive resistance of each material was measured by applying a static load of 25N using a universal testing machine for one minute. Linear dimensional stability was assessed by comparing the diameters of the samples before and after compression using a vernier caliper.

Results: Bona-Bite exhibited the highest compressive resistance with mean values of 0.315±0.066 (6mm), 0.319±0.065 (4mm), and 0.306±0.065 (2mm). It also showed the least linear dimensional change, with mean values of 0.073±0.040 (6mm), 0.105±0.057 (4mm), and 0.080±0.036 (2mm). In comparison, Ramitec and O-Bite demonstrated lower compressive resistance and higher dimensional changes. Statistical analysis revealed significant differences between Bona-Bite and the other materials in both compressive resistance and dimensional stability.

Conclusion: Bona-Bite (polyvinyl siloxane) outperformed Ramitec (polyether) and O-Bite (polyvinyl siloxane) in terms of compressive resistance and dimensional stability. Therefore, Bona-Bite is recommended for use in interocclusal recording to achieve more accurate and reliable prosthetic outcomes.

## Introduction

Prosthetic treatment involves procedures in the absence of a patient and requires articulated casts. An articulator must replicate the patient's jaw motions as closely as possible in order to create an accurate prosthesis [1]. In some circumstances, such as fully edentulous, extremely worn-out conditions, or distal extension edentulous, the casts can be mounted in maximum intercuspation with ease by stabilising them with cast cement following hand articulation. In these situations, however, a medium to articulate is required in order to position the cast in the proper maxillary-mandibular relationship. We refer to this type of medium as an interocclusal recording medium. It is important to accurately capture and transfer the maxillomandibular connection to the articulator through the use of adequate interocclusal recording.

Materials used for interocclusal recording bear similarities to imprint materials that have been altered for handling purposes [1]. Using natural waxes, Phillip Pfaff presented the first interocclusal registrations in 1756 [2]. Numerous interocclusal recording materials were employed, including elastomers, metallic pastes, plaster of Paris, acrylic resins, and waxes [1]. Even today, dental waxes and bite registration pastes containing zinc oxide ethylene are used because they are simple to use, affordable, and require less time. Wax and polyvinyl siloxane are the most often utilised interocclusal record materials in the current situation [2]. A bite registration substance that is optimal should (a) minimise resistance before setting so that the mandible or teeth can move freely during closure, (b) after setting, experience little dimensional change, (c) have the ability to accurately record the incisal and occlusal surfaces, (d) require simpler handling, (e) be biocompatible, and (f) be readily verifiable [3].

Occlusion is one of the critical factors that determines a successful prosthetic treatment, and it demands an accurate positional relationship of the jaws or the dentition. For accurate transfer of the maxillomandibular relation, the interocclusal recording material used should possess dimensional stability and compressive resistance with the increase in time, and not be brittle after setting. Any changes in the dimension of the record causes severe adjustments in the prosthesis as the horizontal and vertical relationship gets altered [4]. Properties such as thickness, storage, and time required in recording the bite affect the force exerted on the record. The clinician must select the appropriate material from the many interocclusal recording materials on the market. The dentist must be aware of the material's composition, properties, manipulation technique, and other factors that may affect the interocclusal recording material. The qualities of bite recording materials have been the subject of numerous studies [2,5].

The objectives of this study were to evaluate and compare the dimensional stability and compressive resistance of three commonly used interocclusal recording materials in India: Ramitec (polyether; 3M ESPE, St. Paul, MN), Bona-Bite (polyvinyl siloxane; DMP Dental, Markopoulo, Greece), and O-Bite (polyvinyl siloxane; DMG Dental, Hamburg, Germany) and to determine the influence of different material thicknesses (2, 4, and 6mm) on the dimensional stability and compressive resistance of the selected interocclusal recording materials.

## Materials and methods

Equipment and materials utilized

This in vitro comparative experimental study utilized a universal testing machine and a vernier caliper for testing. Three types of interocclusal recording materials were employed: Bona-Bite (polyvinyl siloxane), O-Bite (polyvinyl siloxane), and Ramitec (polyether). The materials were categorized into three primary groups based on their type and manufacturer. Group Polyether (PE) included Ramitec with subgroups of 2, 4, and 6mm thicknesses; Group Polyvinyl siloxane A (PVS A) comprised Bona-Bite also subdivided into 2, 4, and 6mm thicknesses; and Group Polyvinyl siloxane B (PVS B) included O-Bite, similarly divided into 2, 4, and 6mm thicknesses (Table 1).

**Table 1:** Materials categorized into three primary groups based on their type and manufacturer

Groups	Materials	Subgroups
Polyether (PE)	Ramitec (3M ESPE, St. Paul, MN)	2mm
		4mm
		6mm
Polyvinyl siloxane A (PVS A)	Bona-Bite (DMP Dental, Markopoulo, Greece)	2mm
		4mm
		6mm
Polyvinyl siloxane B (PVS B)	O-Bite (DMG Dental, Hamburg, Germany)	2mm
		4mm
		6mm

Fabrication of die

**Figure 1:**
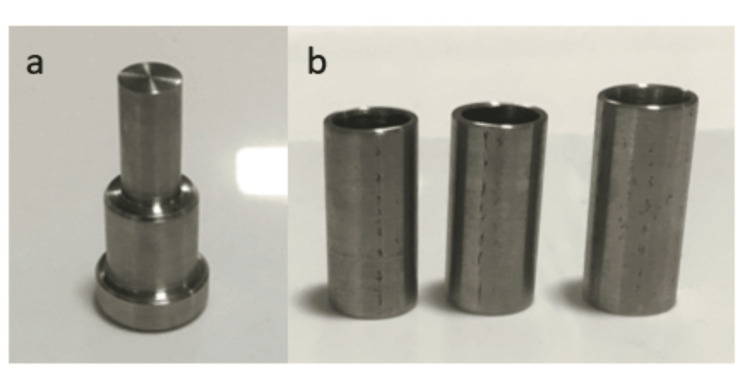
(a) Master cylindrical die; (b) stainless steel hollow die

A master cylindrical die made from stainless steel was fabricated according to the revised American Dental Association specification no. 19. Three additional stainless steel dies, with an internal diameter of 10mm and open at both ends, were precisely machined to fit snugly over the master die (Figures 1a-1b).

**Figure 2:**
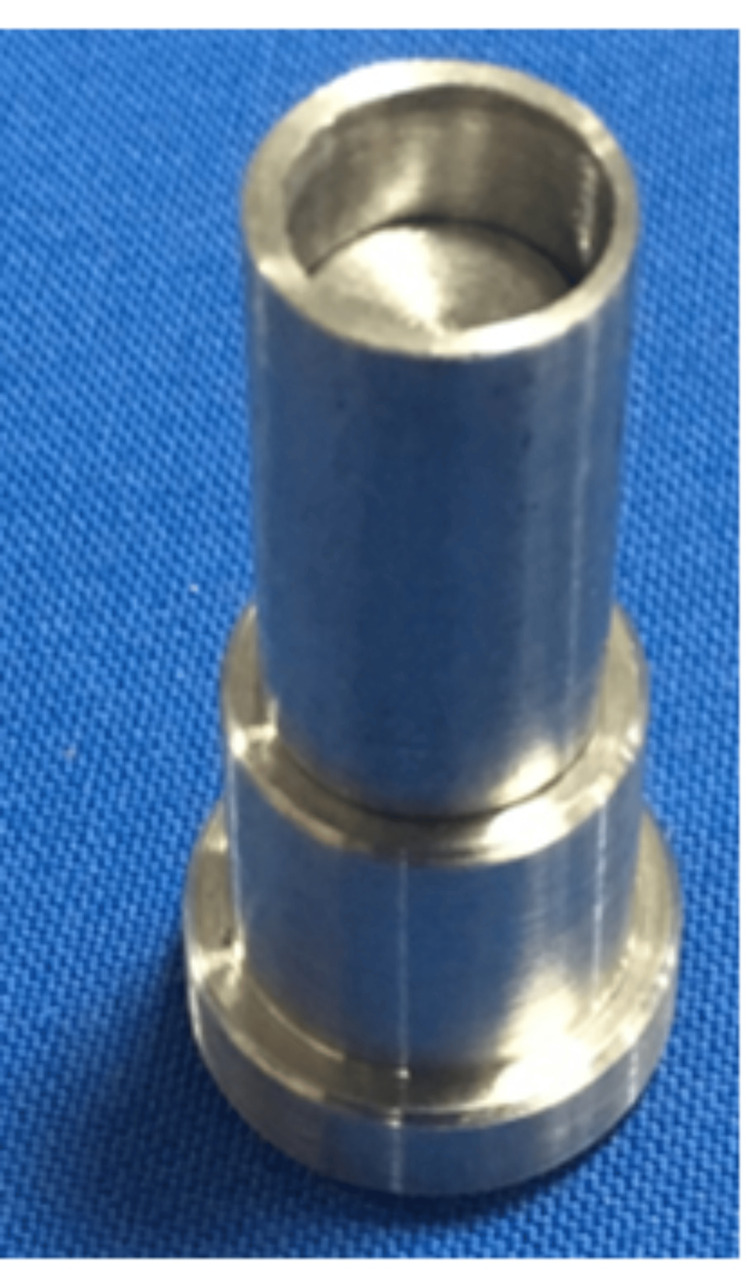
Snugly fit hollow cylindrical die over the metal jig

These dies were hollow cylinders with varying heights to create a height difference of 2, 4, and 6mm when placed on the master die (Figure 2).

**Figure 3:**
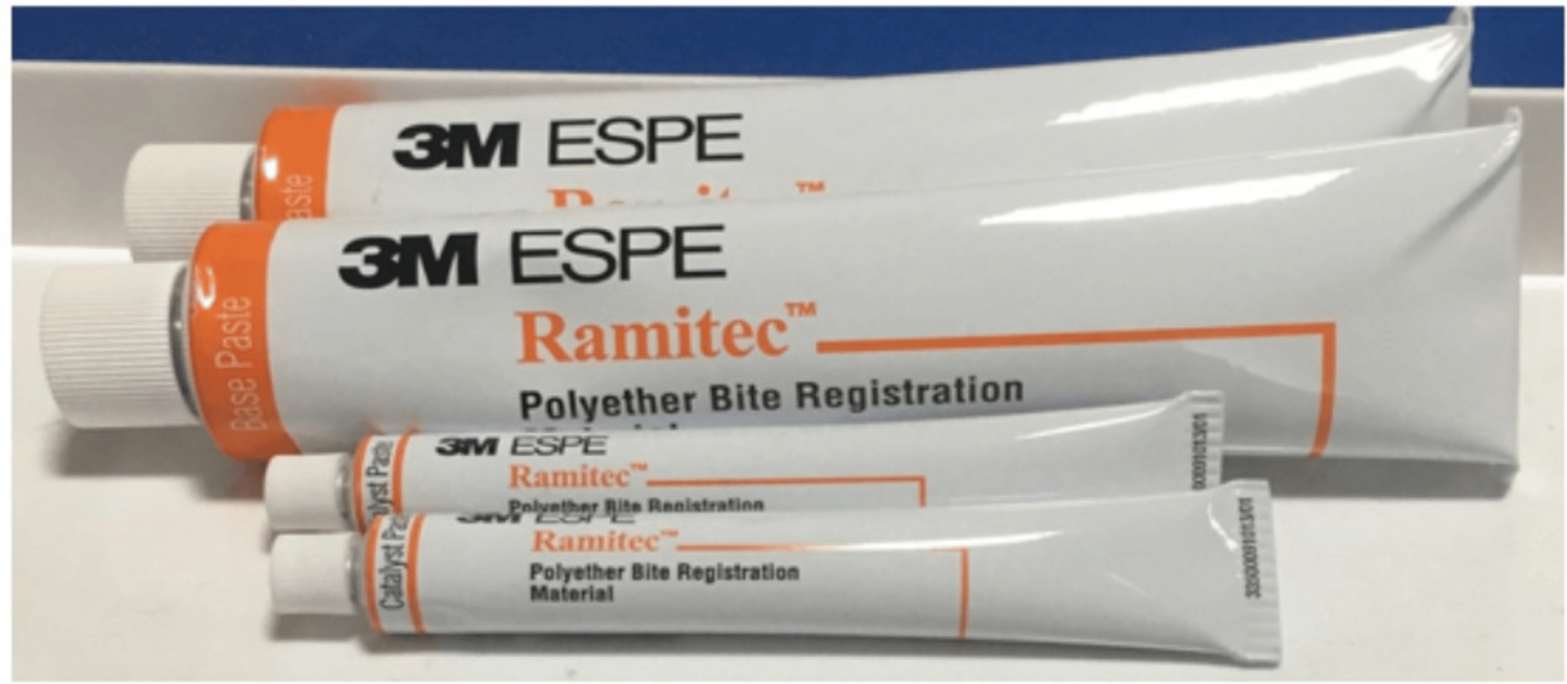
Ramitec

**Figure 4:**
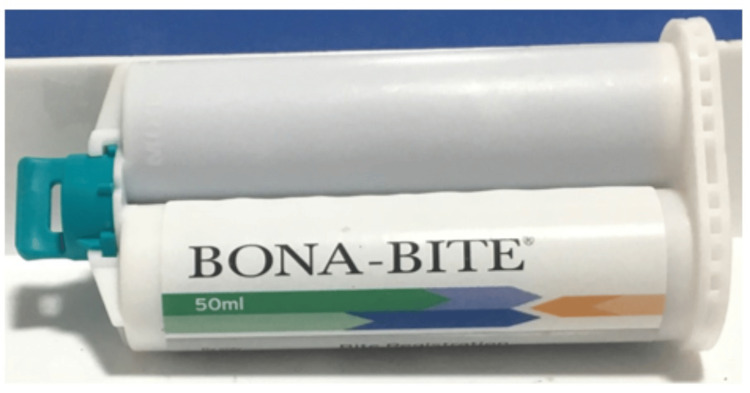
Bona-Bite

**Figure 5:**
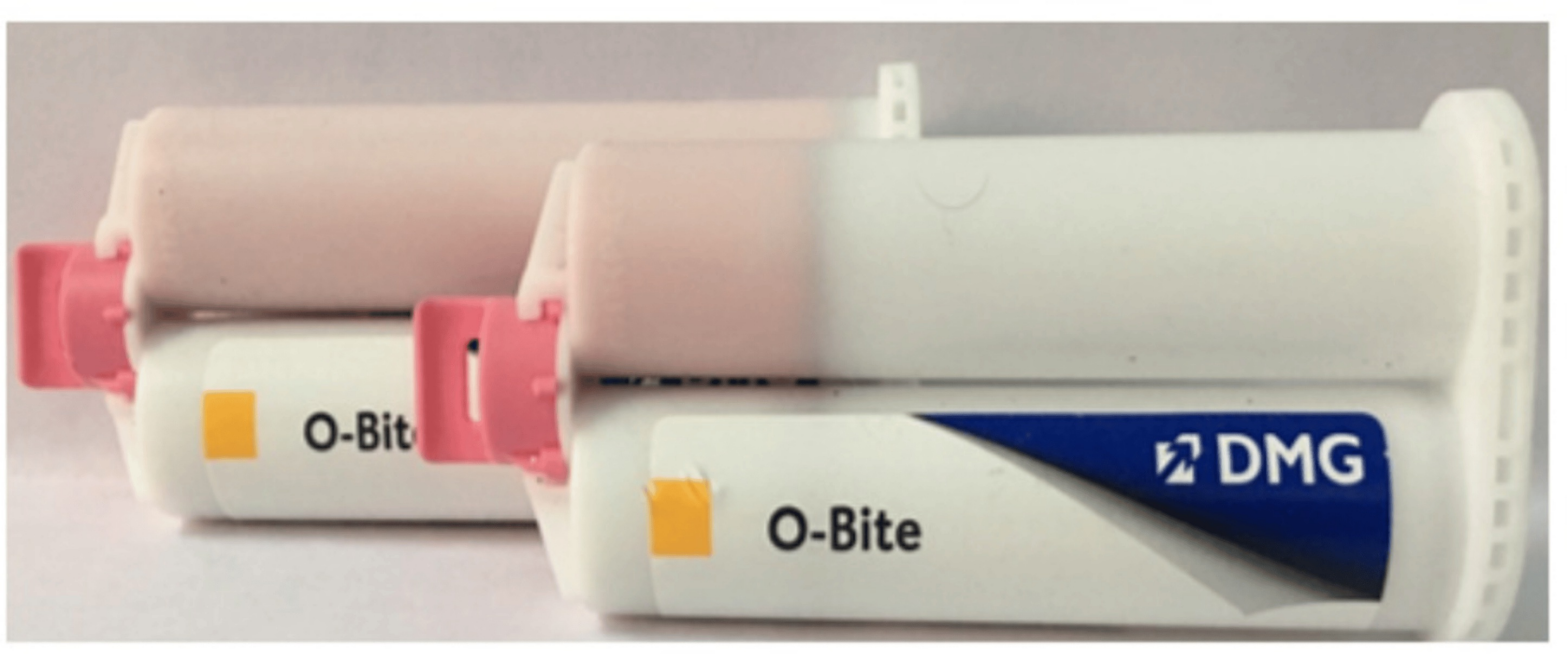
O-Bite

A total of 36 samples were fabricated, with 12 samples each for the thicknesses of 2, 4, and 6mm per material. Thus, 108 samples were created and grouped into three primary categories: PE (Ramitec; Figure 3), PVS A (Bona-Bite; Figure 4), and PVS B (O-Bite; Figure 5), with further subgrouping based on the thicknesses of 2, 4, and 6mm.

Specimen fabrication

To facilitate the removal of the set material, a lubricating agent was applied inside each die. The metallic cylinder was then securely fitted to the metallic base on one side. The respective material was injected into the die, which was then covered with a glass plate. After the material had set, it was removed from the die. Each material was manipulated according to the manufacturer’s instructions. Polyvinyl siloxane materials (Bona-Bite and O-Bite) were provided in auto-mixing cartridges, which were dispensed using a cartridge-loaded dispensing gun with a mixing tip attached. Polyether (Ramitec) was provided in tubes and mixed using a stainless steel spatula in a ratio of 8.3g base paste to 1g catalyst paste until a uniform color and consistency were achieved. The resulting mixture was loaded into a syringe provided by the manufacturer and injected into the die, which was then covered with a glass plate to produce a flat surface. The samples were allowed to set for two to four minutes before being removed from the die. A total of 36 samples were prepared for each material, resulting in 108 samples in total.

Testing of the specimens

**Figure 6:**
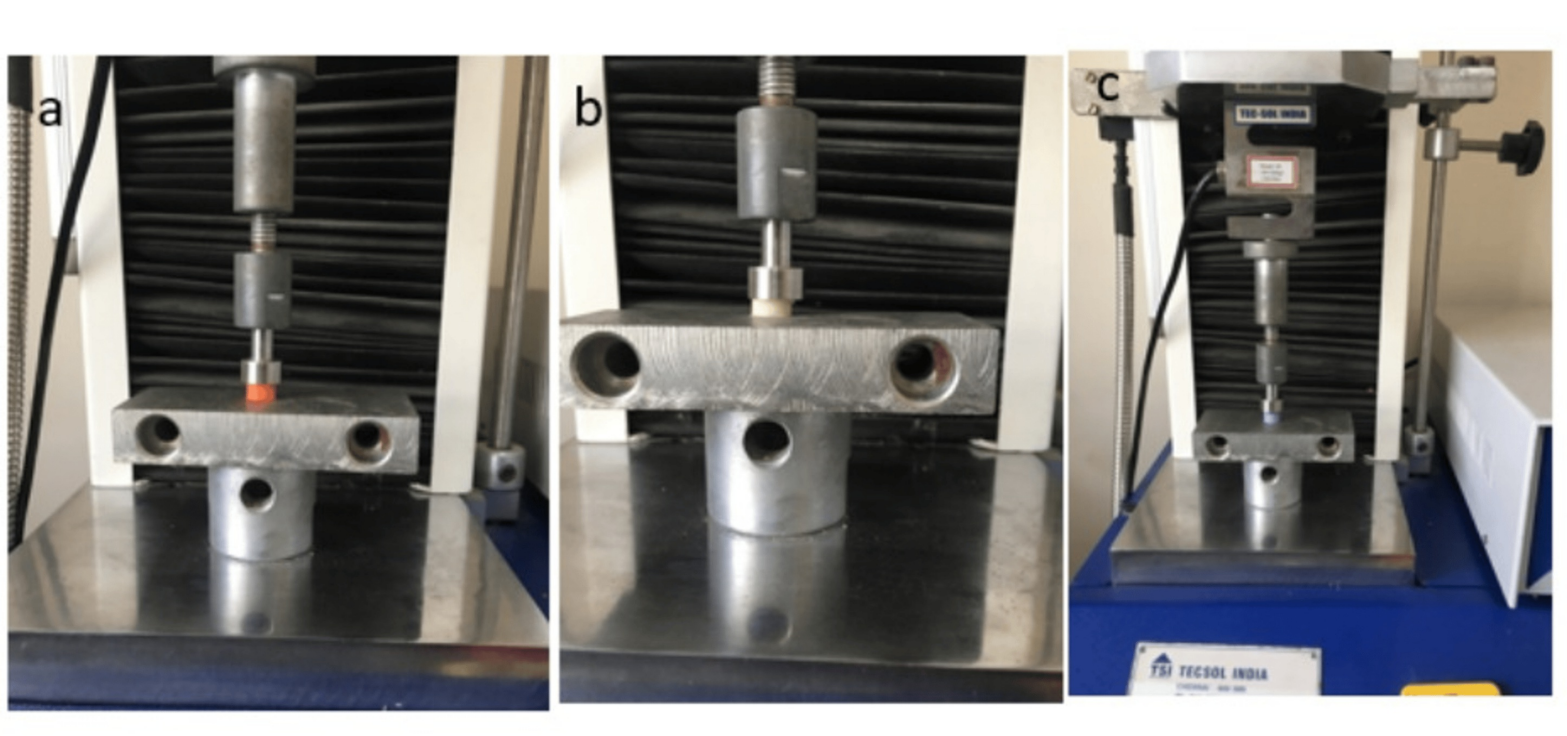
Compression at 25N of (a) O-Bite, (b) Ramitec, and (c) Bona-Bite

The prepared samples were positioned between the plates of a universal testing machine. A constant compressive load of 25N was applied for one minute, with the time recorded using a stopwatch. Before compression, the initial diameter of each sample was measured using a vernier caliper. After applying the 25N load, the resistance to compression was determined by measuring the displacement under the set load after one minute (Figures 6a-6c).

**Figure 7:**
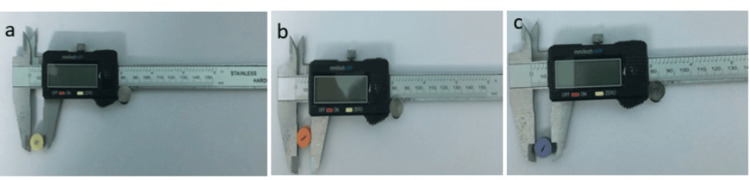
Linear dimensional change measured using a vernier caliper: (a) Ramitec, (b) O-Bite, (c) and Bona-Bite

The linear dimensional stability of the samples was assessed by comparing the diameters measured before and after compression using the vernier caliper (Figures 7a-7c).

## Results

The present study compared the compression distance and linear dimensional changes of three materials, Ramitec, Bona-Bite, and O-Bite, across different thicknesses (6, 4, and 2mm). Ramitec demonstrated consistent compression and low dimensional instability, with a slight dip in compression at 4mm. Bona-Bite showed the least compression and dimensional change, indicating stability, although its variability increased at 4mm. O-Bite exhibited the highest compression and dimensional changes, especially at 4mm, indicating greater instability and variability. These findings highlight the relative performance and stability of these materials under varying conditions.

**Figure 8:**
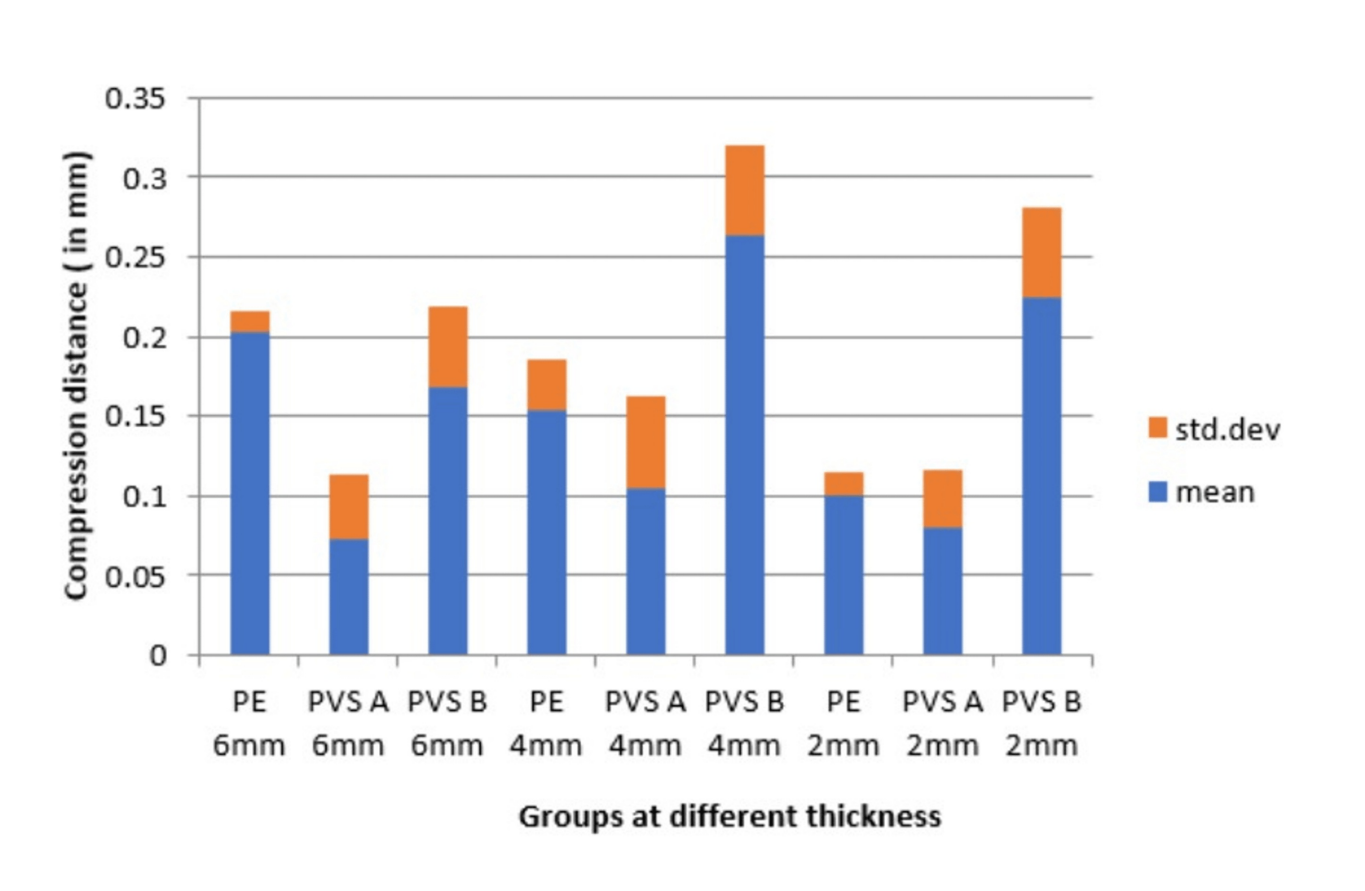
Comparison of compression distance values (in mm) of Group PE, PVS A, and PVS B specimens at 6, 4, and 2mm thicknesses PE: Polyether; PVS A: Polyvinyl siloxane A; PVS B: Polyvinyl siloxane B

The comparison involved the compression distances (mm) for Ramitec, Bona-Bite, and O-Bite at 6, 4, and 2mm thicknesses. Ramitec showed consistent values with a slight dip at 4mm (0.525mm) compared to 6mm (0.597mm) and 2mm (0.594mm). Bona-Bite had the lowest and most stable compression values across all thicknesses (0.306-0.319mm). O-Bite exhibited the highest compression, especially at 4mm (0.758mm), with slightly lower values at 6 and 2mm. Standard deviations and confidence intervals indicated a greater variability for O-Bite, particularly at 4mm, while Ramitec and Bona-Bite were more consistent (Table 2, Figure 8).

**Table 2:** Comparison of compression distance values (in mm) for Ramitec (polyether), Bona-Bite (polyvinyl siloxane), and O-Bite (polyvinyl siloxane) at 6, 4 and 2mm thicknesses p-value less than 0.05, statistically significant

Material	N	Mean	Std. dev.	95% Confidence interval for mean	
				Lower bound	Upper bound
Ramitec 6mm	12	0.597	0.1578	0.497	0.697
Ramitec 4mm	12	0.525	0.159	0.424	0.626
Ramitec 2mm	12	0.594	0.122	0.516	0.671
Bona-Bite 6mm	12	0.315	0.0657	0.273	0.356
Bona-Bite 4mm	12	0.319	0.065	0.277	0.360
Bona-Bite 2mm	12	0.306	0.065	0.265	0.348
O-Bite 6mm	12	0.625	0.0945	0.564	0.685
O-Bite 4mm	12	0.758	0.133	0.673	0.843
O-Bite 2mm	12	0.670	0.112	0.598	0.741

**Figure 9:**
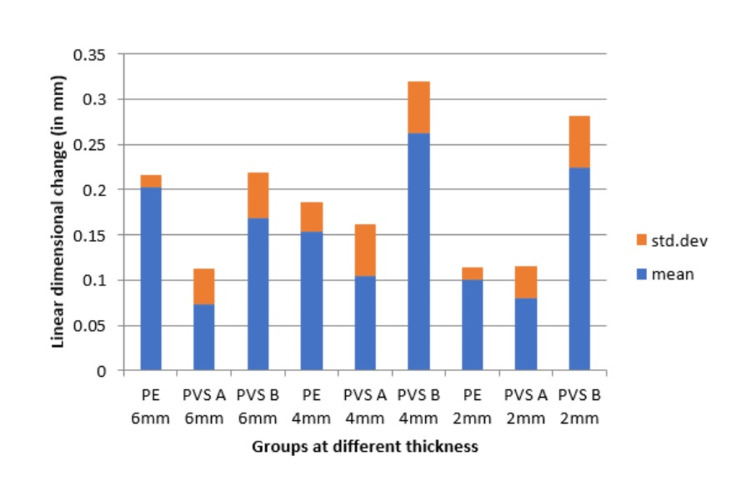
Comparison of linear dimensional changes (in mm) of Group PE, PVS A and PVS B specimens at 6, 4, and 2mm thicknesses PE: Polyether; PVS A: Polyvinyl siloxane A; PVS B: Polyvinyl siloxane B

Table 3 presents data on the linear dimensional changes of three materials, Ramitec, Bona-Bite, and O-Bite, measured at different thicknesses (6, 4, and 2mm). Ramitec exhibited the lowest dimensional change at the 2mm thickness (0.100mm) and a relatively low change at 4 and 6mm (0.154 and 0.203mm, respectively), indicating minimal dimensional instability. Bona-Bite showed the smallest changes across all thicknesses, with the lowest being at 6mm (0.073mm) and slightly higher values at 2 and 4mm. However, its data was more variable, particularly at 4mm. O-Bite displayed the highest dimensional changes, especially at 4 and 2mm thicknesses (0.263 and 0.225mm, respectively), suggesting greater dimensional instability compared to Ramitec and Bona-Bite. The confidence intervals suggested varying degrees of precision in the mean estimates, with Bona-Bite at 4mm and O-Bite at 4mm having the most variability (Table 3, Figure 9).

**Table 3:** Comparison of linear dimensional changes (in mm) of Group PE, PVS A and PVS B specimens at 6, 4, and 2mm thicknesses p-value less than 0.05, statistically significant

Materials	N	Mean	Std. dev.	95% Confidence intervals for mean		
				Lower bound	Upper bound	
Ramitec 6mm	12	0.203	0.0136	0.194		0.211
Ramitec 4mm	12	0.154	0.326	0.133		0.174
Ramitec 2mm	12	0.100	0.014	0.091		0.109
Bona-Bite 6mm	12	0.073	0.0403	0.048		0.099
Bona-Bite 4mm	12	0.105	0.576	0.069		0.142
Bona-Bite 2mm	12	0.080	0.036	0.057		0.103
O-Bite 6mm	12	0.168	0.0514	0.135		0.200
O-Bite 4mm	12	0.263	0.574	0.226		0.299
O-Bite 2mm	12	0.225	0.056	0.182		0.261

## Discussion

The main goals of occlusal rehabilitation are to achieve the best possible oral health, functional effectiveness, comfort, and aesthetics. In order to restore function, comfort, and aesthetics during oral rehabilitation, recording maxillomandibular connections is crucial. The relationship between the maxillary and mandibular jaws is recorded using the interocclusal record, which is then transferred to a dental articulator to match the patient's mouth. Maxillomandibular associations can be recorded using a variety of techniques, including graphic, functional, cephalometric, and direct interocclusal records [6]. The maxillomandibular connection of dentulous castings is typically recorded using direct interocclusal records. The initially soft recording material hardens as it fills in the crevices between teeth. After that, the casts and the set material are placed onto an articulator [7]. For castings on an articulator to transfer the jaw relationship to the mouth, an interocclusal recording medium needs to be precise and dimensionally stable [8].

Yurkstas and Kapur evaluated the duplicity of the centric relation using the wax record and intra-oral tracers [9]. They stated that variables such as the amount of pressure applied in making the record, the size of the alveolar ridge, consistency of wax, and location and angulation of the central bearing point and plate significantly influenced the duplicity of centric relation records. The amount of pressure exerted was found to adversely affect the duplicity of the record. Interocclusal recording materials such as polyether and polyvinylsiloxane have high flow and least resistance to compression where the duplicity of the record is least affected. The fundamental guidelines for choosing interocclusal records were provided by Freilich et al. [10]. They indicated that reliable and repeatable recording of the maxillomandibular connection requires a tripod of vertical support with adequate horizontal stability between the two casts.

On researching the impact of storage, Lassila and McCabe found that moisture caused significant expansion, necessitating appropriate packaging during storage and transit, and that the elastomeric interocclusal recording material remained dimensionally stable for a long period [11,12]. A variety of physical characteristics, including a low viscosity level, low resistance to closure, ease of use, sufficient working time, accuracy in detail, quick hardening, biocompatibility, and verifiability, have been shown to be desirable for an ideal recording material, even though none of the materials fully satisfy all of the requirements.

The most ideal properties of the interocclusal recording medium are resistance to compression and linear dimensional change. In order to prevent restorative errors, which arise from a discrepancy between the mounted cast as well as the intra-oral relationship of the teeth, the material should be sufficiently rigid to withstand distortion caused by the weight of the dental casts, the articulator's components, or other means used to stabilise the casts during the mounting procedure [13]. Cast articulation may be delayed for a number of reasons. For example, the addition of plasticisers and catalysts to polyether and polyvinyl siloxane interocclusal recording materials to improve handling characteristics may have an impact on the material's dimensional characteristics, which in turn may affect the exact articulation of the casts [14].

The purpose of this in vitro investigation was to evaluate the linear dimensional change and compressive resistance of three distinct interocclusal recording materials under compressive pressure. The materials selected for this investigation were two polyvinyl siloxane materials (Bona-Bite and O-Bite) and one polyether material (Ramitec), which are the most widely accessible interocclusal recording materials in India. Three subgroups were created from the 15 specimens in each group, according to the thickness of the interocclusal recording material (6, 4, and 2mm). The samples were created in compliance with the manufacturer's guidelines. Every sample was put inside a universal testing machine that applied a static pressure of 25N for a duration of one minute. Rubber bands are frequently used to stabilise the opposing casts together during the mounting process [15].

Chandu et al. also utilized this approach in a study where the maximum force was applied by securing the maxillary and mandibular casts on an articulator using a single standard no. 19 rubber band, in combination with the weight of the casts over the recording medium [16]. The compressive distance on the universal testing machine was used to measure the readings for resistance to compression, and a vernier caliper was used to determine the difference in the dimensional change prior to and after compression under the universal testing machine to measure the values for linear dimensional stability. These values were statistically analysed using one-way ANOVA for compressive resistance and dimensional changes between the groups and within the groups and the results were statistically significant (p=0.001), as shown in Tables 1-2.

When the 6mm thickness was tested using the Bonferroni method, no statistically significant difference was found between PE/Ramitec and PVS B/O-Bite, but a statistically significant difference was observed between PVS A/Bona-Bite and PVS B/O-Bite. At 4mm thickness, there was a statistically significant difference between PE/Ramitec and PVS A/Bona-Bite, as well as between both PVS A/Bona-Bite and PVS B/O-Bite, and PE/Ramitec and PVS B/O-Bite. For the 2mm thickness, a statistically significant difference was noted between PE/Ramitec and PVS A/Bona-Bite, and between PVS A/Bona-Bite and PVS B/O-Bite, but there was no significant difference between PE/Ramitec and PVS B/O-Bite.

On using a one-way ANOVA within the group, Ramitec's statistical analysis revealed no statistically significant difference between 6mm and 2mm, although there was a statistically significant difference between 4mm and 2mm. For the Bona-Bite interocclusal registration material, there was a statistically significant difference between 6mm and 4mm, as well as between 4mm and 2mm. However, there was no statistically significant difference between 6mm and 4mm. In the case of O-Bite, no significant difference was observed between 6mm and 2mm, although there was a statistically significant difference between 4mm and 2mm.

The findings of this in vitro investigation demonstrate that, when compared to Ramitec as well as O-Bite interocclusal registration materials, the Bona-Bite interocclusal recording material, with thicknesses of 6, 4, and 2mm, demonstrated a higher resistance to compression, with mean and standard deviation values of 0.315±0.066, 0.319±0.065, and 0.306±0.065, respectively. According to the findings of a study put forth by Tejo et al., the polyether interocclusal recording materials was more resilient to compression than polyvinyl siloxane [15]. Our findings aligned with those of Breeding and Dixon and Nagrath et al., which demonstrated that polyvinylsiloxane outperformed polyether (Ramitec) interocclusal recording materials in terms of resistance to compression and dimensional stability [3,17,18].

According to Wilson and Banerjee, there are other aspects besides the physical characteristics of an interocclusal recording material that affect its accuracy and reproducibility. These factors include the patient, the operator's skill level, and the recording technique [6,19]. Patient-guided procedures are less effective than guiding the patient to a retruded position.

The strengths of this study lie in its comparative analysis and controlled methodology. By evaluating three commonly used interocclusal recording materials (Ramitec, Bona-Bite, and O-Bite), the study provided a comprehensive understanding of their relative performance in terms of dimensional stability and compressive resistance. The use of a master stainless steel die and consistent sample preparation ensured uniformity, reducing variability and enhancing the reliability of the findings. Additionally, including different types of polyvinyl siloxane and polyether materials improved the generalizability of the results, and the statistical analysis provided a quantitative basis for assessing differences between the materials, adding rigor to the conclusions.

This study also has limitations. Being conducted in vitro, it may not fully replicate the complex conditions present in the oral cavity, which could impact the clinical applicability of the findings. The study included only three specific interocclusal recording materials, limiting the scope of comparison, and additional materials could provide more comprehensive insights. Furthermore, the compressive resistance was tested under a single static load of 25N for one minute, which does not capture the dynamic forces encountered in clinical settings. Lastly, only three specific thicknesses (2, 4, and 6mm) were tested, and evaluating a wider range of thicknesses could reveal more detailed performance characteristics under different clinical scenarios.

## Conclusions

This in vitro study aimed to compare the compressive resistance and dimensional stability of three different interocclusal recording materials, one polyether (Ramitec) and two polyvinyl siloxane (Bona-Bite and O-Bite) at varying thicknesses (2, 4, and 6mm). The results demonstrated that Bona-Bite exhibited the greatest resistance to compression across all thicknesses, indicating its superior ability to maintain dimensional stability under load. This suggests that Bona-Bite may be the preferred material for situations where high compressive strength and minimal dimensional change are crucial, particularly in complex prosthetic treatments requiring precise maxillomandibular relationships.

The study confirmed that while polyether materials like Ramitec have their merits, polyvinyl siloxane materials, especially Bona-Bite, are better suited for clinical applications where compressive resistance and dimensional stability are prioritized. The findings support the use of Bona-Bite as a reliable interocclusal recording material, ensuring an accurate transfer of maxillomandibular relationships to the articulator, which is essential for successful prosthetic outcomes.

## References

[1] Dua P, Gupta SH, Ramachandran S, Sandhu HS (2007). Evaluation of four elastomeric interocclusal recording materials. Med J Armed Forces India.

[2] Gurav SV, Khanna TS, Nandeeshwar DB (2015). Comparison of the accuracy and dimensional stability of interocclusal recording materials: an in-vitro study. Int J Sci Res Publ.

[3] Breeding LC, Dixon DL (1992). Compression resistance of four interocclusal recording materials. J Prosthet Dent.

[4] Campos AA, Nathanson D (1999). Compressibility of two polyvinyl siloxane interocclusal record materials and its effect on mounted cast relationships. J Prosthet Dent.

[5] Malone WP, Koth Koth, DL DL, Cavazos E, Kaiser DA, Morgano SM, Tylman SD (1989). Tylman's Theory and Practice of Fixed Prosthodontics, 8th Edition. Tylman’s theory and practice of fixed prosthodontics, 8th edition.

[6] Wilson PH, Banerjee A (2004). Recording the retruded contact position: a review of clinical techniques. Br Dent J.

[7] Hembree JH Jr, Nunez LJ (1974). Effect of moisture on polyether impression materials. J Am Dent Assoc.

[8] Skurnik H (1969). Accurate interocclusal records. J Prosthet Dent.

[9] Yurkstas AA, Kapur KK (2005). Factors influencing centric relation records in edentulous mouths. J Prosthet Dent.

[10] Freilich MA, Altieri JV, Wahle JJ (1992). Principles of selecting interocclusal records for articulation of dentate and partially dentate casts. J Prosthet Dent.

[11] Lassila V, McCabe JF (1985). Properties of interocclusal registration materials. J Prosthet Dent.

[12] Giri TK, Banerjee S, Banerjee TN, Mandal BC, Dongre P, Khanna P, Giri D (2024). A comparative study to evaluate the linear dimensional change and compression resistance of four elastomeric interocclusal recording materials: an in vitro study. J Pharm Bioallied Sci.

[13] Michalakis KX, Pissiotis A, Anastasiadou V, Kapari D (2004). An experimental study on particular physical properties of several interocclusal recording media. Part II: Linear dimensional change and accompanying weight change. J Prosthodont.

[14] Michalakis KX, Pissiotis A, Anastasiadou V, Kapari D (2004). An experimental study on particular physical properties of several interocclusal recording media. Part III: resistance to compression after setting. J Prosthodont.

[15] Tejo SK, Kumar AG, Kattimani VS, Desai PD, Nalla S, Chaitanya KK (2012). A comparative evaluation of dimensional stability of three types of interocclusal recording materials-an in-vitro multi-centre study. Head Face Med.

[16] Chandu GS, Khan MF, Mishra SK, Asnani P (2015). Evaluation and comparison of resistance to compression of various interocclusal recording media: an in vitro study. J Int Oral Health.

[17] Karthikeyan K, Annapurni H (2007). Comparative evaluation of dimensional stability of three types of interocclusal recording materials: an in vitro study. J Prosthet Dent.

[18] Nagrath R, Lahori M, Kumar V, Gupta V (2014). A comparative study to evaluate the compression resistance of different interocclusal recording materials: an in vitro study. J Indian Prosthodont Soc.

[19] Millstein PL, Clark RE (1981). Differential accuracy of silicone-body and self-curing resin interocclusal records and associated weight loss. J Prosthet Dent.

